# The association between paternal PTSD and child mental health: a systematic literature review

**DOI:** 10.1186/s13034-026-01073-w

**Published:** 2026-05-17

**Authors:** Liquori L. Etheridge, Sungseek Moon, Aynsley H. Scheffert, Christina Chan-Park, Danny Clark

**Affiliations:** 1 Social Work, Baylor University, Waco, USA; 2 University of Iowa, Iowa City, USA

**Keywords:** Paternal, Father, PTSD, Children, Adolescent, Teen, Anxiety, Depression, Hyper-activity attention-deficit/hyperactivity disorder (ADHD), Behavior disorders

## Abstract

Fathers play a crucial role in their children’s psychosocial development, positively influencing emotional, cognitive, and social competencies. Paternal Post-Traumatic Stress Disorder (PTSD) can disrupt this dynamic, potentially compromising the psychological well-being and developmental outcomes of their offspring. This systematic review aims to investigate the relationship between paternal PTSD and the prevalence of psychopathology in children and adolescents, providing a comprehensive understanding of how trauma experienced by fathers may influence their children’s mental health. A systematic search of PsycINFO, PubMed, Embase, Web of Science, and PILOTS yielded only six studies that met the inclusion criteria. Findings indicate a significant association between paternal PTSD and increased risk of depression, anxiety, and behavioral disorders in children and adolescents. Furthermore, the results suggest that maternal mental health status may moderate the relationship between paternal PTSD and offspring mental health outcomes. Future research should explore the mechanisms of trauma transmission from fathers to children, the broader impact of paternal PTSD on family dynamics, and the specific role of maternal mental health in this context.

## Introduction

Childhood mental health is a pressing public health concern, with recent estimates i, ndicating that 1 in 5 children ages three to 17 in the United States experience a mental, emotional, or behavioral disorder [87]. The presence of a parent with a mental health condition significantly increases the risk of these disorders, with studies showing that children of parents with psychiatric symptoms are at a greater likelihood of developing their own mental health conditions [91, 98]. For example, a study examining children referred to a psychiatric outpatient clinic found that 24% of mothers and fathers reported at least subclinical symptoms of either internalizing or externalizing behaviors [91]. Moreover, the National Survey of Children’s Health conducted between 2016 and 2018 revealed an estimated 7.2% of children had at least one caregiver with poor mental health, underscoring the critical link between parental mental health and child outcomes [32].

Despite this evidence, there exists a significant gap in understanding the specific role of paternal mental health in children’s development, as the majority of the literature has focused on maternal mental health [80, 85]. This oversight raises pressing questions about how paternal mental health conditions, particularly trauma and PTSD, influence child outcomes and highlights the urgent need for more comprehensive research in this area. As societal norms evolve and family dynamics shift, it becomes increasingly important to explore the paternal influence on child biopsychosocial development. Yet, studies examining this influence have primarily assessed the child-maternal relationship and impact on child psychological, emotional, and behavioral development, leaving the role of father underexplored [80, 85]. To quantify the gender disparity in parent-child research, in 1992, Phares and Compas found that 48% of the studies included mothers only and 1% included fathers only. This trend has continued. Parent et al. [51] reviewed 1,048 studies published from 2005 to 2015 and identified 37% of the studies involved mothers only, 23% involved mothers and fathers and the proportion of studies focused on fathers only remained at 1%. This imbalance underscores the critical need to further investigate the impact of paternal mental health on child development and mental health.

While studies on maternal perinatal mental health are more prevalent compared to paternal perinatal mental health, researchers are increasing their focus with respect to paternal perinatal depression and anxiety. For instance, a longitudinal study focused on paternal perinatal mental health found that one in 20 new fathers experienced depression following the birth of their child [86]. The increased rates of paternal perinatal depression were supported by evidence indicating that 8% of men experienced depression during pregnancy and up to a year postpartum, twice the rate compared to the general population of men [11]. Research evidence demonstrates that children of fathers who experience perinatal depression have increased behavioral problems, hyperactivity, lower psychosocial functioning, and increased suicidal ideations [56, 60]. An additional study that explored the impact of fatherhood on paternal mental health found that 5–10% of fathers experienced depression and anxiety for up to one year following the child’s birth [82]. Although research focused on the impact of paternal mood disorders (including anxiety and depression) on offspring is increasing, the gap in research analyzing the effects of paternal trauma and PTSD on child offspring warrants increased attention.

Many of the studies exploring the relationship between paternal PTSD and its impact on children primarily focus on military service members and/or military veterans. Epidemiological and clinical studies demonstrate that combat-related PTSD is associated with other co-morbid psychiatric disorders, and veterans with PTSD also report higher rates of interpersonal conflict [36]. The occurrence of PTSD with military fathers was not only associated with increased family dysfunction [89]; children and spouses of Vietnam-veteran fathers with PTSD reported lower self-esteem, as well as emotional and psychiatric disturbances [47].

Symptoms of PTSD are differentiated into four symptom clusters: intrusive symptoms (five), avoidance symptoms (two), negative alterations in cognition and mood (NACM) symptoms (seven), and arousal symptoms (six). Of these paternal PTSD symptom clusters, avoidant symptoms are believed to be the most impactful on the family system [26]. A diagnosis of PTSD requires the presence of at least one intrusion, one avoidance, two NACM, and two arousal symptoms [4]. In addition to symptomology, functional impairment is required to meet criteria for a diagnosis of PTSD [4]. Examples of functional impairment include decreased functioning at home, school, or work, detachment from loved ones, and difficulty sleeping [59]. PTSD symptoms experienced by parents also impacted several parenting domains and parental well-being. PTSD in parents, both mothers and fathers, has been associated with reduced parental satisfaction, increased parental stress, changes in parent-child relationships, and other parenting practices [15].

However, there was notable variability in determining the specific relationships between these symptom clusters and various aspects of parental well-being, especially between maternal and paternal presentations [17, 79, 101]. For example, a study by Samper et al. [101] revealed that avoidance symptoms were correlated with negative parenting behaviors in fathers; no significant association was found between hyperarousal or reexperiencing symptoms and parenting outcomes. By contrast, Berz et al. [79] reported different findings among mothers. They found a correlation between a reduction in parenting satisfaction and both avoidance and hyperarousal symptoms. They did not find a significant association with reexperiencing symptoms. The association between maternal PTSD and negative parenting satisfaction was challenged by a study conducted by Creech et al. [18]. Although researchers identified a relationship between PTSD symptoms among women who deployed in support of combat operations and negative family and intimate relationship satisfaction,parenting satisfaction was significantly and negatively associated with post-deployment stress only and not PTSD symptoms. However, these studies do not provide any insight whether the child outcomes are impacted differently if the active-duty parent was the mother or the father. When such distinction is made in veteran samples (e.g., 84, 94]) the results appear contradictory and suggest that female veterans were more likely to endorse adverse child functioning to include behavior, academic performance, peer relationships, emotional problems, and physical problems compared with male veterans.

An additional gap identified in research examining the association of parental PTSD and impact on family has been the use of self-reported scales. Christie et al. [15], in a review of literature on parental PTSD and associations with parent-child relationships, noted mixed results from studies assessing parent and child bonding potentially linked to the reliance on self-report measures. These self-reported measures of the parent’s symptoms and perspectives may have been influenced by the parent’s mental health, demonstrating the need to conduct additional studies. The overall mental health of the parent can significantly influence responses on self-reports designed to assess the child’s overall mental and behavioral health functioning. When assessing child psychological symptoms, structured interviews and assessments conducted by mental health providers can increase the reliability of findings. Incorporating observational approaches will ameliorate potential bias while still providing insight into parental lived experience and the impact of psychopathology on child–paternal relationships [15]. Further gaps in research examining the association between paternal PTSD and child psychopathology identified a disproportionate number of studies that used male military veterans as the primary researched population [25, 31, 42]. The predominant use of military veteran fathers may have impacted the generalizability of these studies.

Moreover, a significant amount of research assessing the relationship between paternal PTSD and offspring psychopathology focused on adult children [2]. Studies examining the association of paternal parenting exposed to trauma and the impact on offspring focused on adult offspring of WWII ex-POWs [2]. Adult offspring reported that their fathers were uncaring about their emotional needs during childhood [81]. Another study examining the impact of paternal trauma of Holocaust survivors on adult children identified the relationship between intrusive memories experienced by the father and increased loneliness and emotional distance (Wiseman 2008). A 23-year-long longitudinal study examining the transmission of paternal trauma to children also assessed adult offspring of ex-POW survivors following the Yom Kippur War [2]. The study found increased rates of PTSD and higher levels of attachment insecurities compared to offspring of ex-POWs without PTSD.

Although these studies demonstrate a relationship between paternal trauma and impact on offspring, the focus on adult children instead of minor children and adolescents prevented researchers from substantiating and generalizing findings to non-adult children. Further exploration of identified research gaps and or inconsistent findings in the literature include detecting the most prevalent PTSD symptom clusters associated with adverse child mental and behavioral health outcomes; identifying articles for inclusion that focus on non-military fathers; focusing specifically on the impact of paternal PTSD on non-adult children; and determining if findings within the literature support the relationship between paternal PTSD and offspring–child psychopathology were associated with self-reports compared to observational child assessments.

### Current study

This study focused on research gaps and inconsistencies in literature. This systematic review then synthesized identified research questions and concepts related to the impact paternal PTSD has on the mental and behavioral well-being of their offspring. The systematic review was conducted by identifying research literature focused on answering the following defined research questions: (1) Are children of fathers diagnosed with PTSD or stress-related disorder at increased risk for adverse mental and behavioral health outcomes? (2) Are specific PTSD symptom clusters experienced by fathers associated with increased risk of child psychopathology? (3) Are there gender differences in child psychological and behavioral health outcomes? (4) Does the use of child-observational studies instead of parental child assessments meaningfully affect the results of this research focused on the impact of paternal PTSD on child offspring?

## Methods

The protocol for this systematic review was registered on PROSPERO and can be accessed at https://www.crd.york.ac.uk/prospero/display_record.php?RecordID=501333. We followed the Preferred Reporting Items for Systematic Reviews and Meta-Analyses (PRISMA) reporting system to complete the systematic review. The following eight common steps were used in accordance with PRISMA guidelines: (1) formulating the research problem; (2) developing and validating the review protocol; (3) searching the literature; (4) screening for inclusion; (5) assessing quality; (6) extracting data; (7) analyzing and synthesizing data; and (8) reporting the findings [100]. The Preferred Reporting Items for Systematic Review and Meta-Analyses Protocol (PRISMA-P) was used to provide evidenced-based resources, data sources, eligibility criteria, screening selection, data extraction, and data analyses evidence [93]. The PRISMA-P model was used to address reporting biases, or the selection of research findings based on the results and selection bias that occur when a systematic review does not identify all available data on a topic (Drucker et al. 2016).

This systematic review identified empirical evidence from research articles that met pre-established inclusion criteria. See Table 1 Database Search Strategy for additional details regarding search. The systematic review demonstrated that the social science field needs to further expand the field of knowledge associated with the relationship between paternal PTSD and impact on child psychopathology [97]. The literature search included backward searches to identify relevant work cited by the articles [99] and forward searches to find all articles that cited the articles reviewed [99].

**Table 1:** Database search strategy

Database	search
PsycInfo	( ( ( "adjustment disorders" OR "adjustment disorder" ) OR ( Anxiety OR DE "Anxiety Disorders" OR DE "Castration Anxiety" OR DE "Generalized Anxiety Disorder" OR DE "Obsessive Compulsive Disorder" OR DE "Panic Attack" OR DE "Panic Disorder" OR DE "Phobias" OR DE "Separation Anxiety Disorder" OR DE "Trichotillomania" OR DE "Anxiety" OR DE "Anxiety Sensitivity" OR DE "Climate Anxiety" OR DE "Computer Anxiety" OR DE "Death Anxiety" OR DE "Health Anxiety" OR DE "Mathematics Anxiety" OR DE "Performance Anxiety" OR DE "Social Anxiety" OR DE "Speech Anxiety" OR DE "Test Anxiety" OR DE "Travel Anxiety" ) OR depression OR ( attention deficit disorder* OR DE "Attention Deficit Disorder" OR DE "Attention Deficit Disorder with Hyperactivity" OR ADHD ) OR ( ( DE "Behavior Disorders" OR DE "Conduct Disorder" OR DE "Disruptive Behavior Disorders" OR DE "Impulse Control Disorders" OR DE "Kleptomania" OR DE "Oppositional Defiant Disorder" OR DE "Pyromania" OR DE "Self-Destructive Behavior" ) OR ( "behavior disorder" OR "behavior disorders" OR "behaviour disorder" OR "behaviour disorders" ) ) ) AND ( teen* OR adolescen* OR youth OR juvenile OR child* ) ) AND ( ( PTSD OR post traumatic stress disorder OR Posttraumatic Stress Disorder OR post-traumatic Stress Disorder ) AND ( father OR parent* OR patern* OR DE "Parents" OR DE "Parental Occupation" OR DE "Parental Characteristics" OR DE "Parent Educational Background" OR DE "Parental Attitudes" OR DE "Parental Investment" OR DE "Parental Occupation" OR DE "Parental Role" OR DE "Parenting Style" OR DE "Permissive Parenting" ) )
PsycInfo	( military OR veteran* OR soldier* OR armed forces OR army OR navy OR air force OR marine* OR deploy* ) AND ( ( ( ( "adjustment disorders" OR "adjustment disorder" ) OR ( Anxiety OR DE "Anxiety Disorders" OR DE "Castration Anxiety" OR DE "Generalized Anxiety Disorder" OR DE "Obsessive Compulsive Disorder" OR DE "Panic Attack" OR DE "Panic Disorder" OR DE "Phobias" OR DE "Separation Anxiety Disorder" OR DE "Trichotillomania" OR DE "Anxiety" OR DE "Anxiety Sensitivity" OR DE "Climate Anxiety" OR DE "Computer Anxiety" OR DE "Death Anxiety" OR DE "Health Anxiety" OR DE "Mathematics Anxiety" OR DE "Performance Anxiety" OR DE "Social Anxiety" OR DE "Speech Anxiety" OR DE "Test Anxiety" OR DE "Travel Anxiety" ) OR depression OR ( attention deficit disorder OR ( DE "Attention Deficit Disorder" OR DE "Attention Deficit Disorder with Hyperactivity" ) OR ( ADHD ) ) OR ( ( DE "Behavior Disorders" OR DE "Conduct Disorder" OR DE "Disruptive Behavior Disorders" OR DE "Impulse Control Disorders" OR DE "Kleptomania" OR DE "Oppositional Defiant Disorder" OR DE "Pyromania" OR DE "Self-Destructive Behavior" ) OR ( "behavior disorder" OR "behavior disorders" OR "behaviour disorder" OR "behaviour disorders" ) ) ) AND ( teen* OR adolescen* OR youth OR juvenile OR child* ) ) AND ( ( PTSD OR post traumatic stress disorder OR Posttraumatic Stress Disorder OR post-traumatic Stress Disorder ) AND ( father OR parent* OR DE "Parents" OR DE "Parental Occupation" OR DE "Parental Characteristics" OR DE "Parent Educational Background" OR DE "Parental Attitudes" OR DE "Parental Investment" OR DE "Parental Occupation" OR DE "Parental Role" OR DE "Parenting Style" OR DE "Permissive Parenting" ) ) )
Embase	('adjustment disorder*' OR depression OR 'depression'/exp OR anxiety OR 'anxiety'/exp OR 'anxiety disorder'/exp OR 'attention deficit disorder' OR adhd OR 'behavio*r disorder*' OR 'behavior disorder'/exp) AND ('juvenile'/exp OR child* OR teen* OR adolescen* OR youth* OR juvenile*) AND ('post traumatic stress disorder'/exp OR ((posttraumatic OR (post AND traumatic)) AND stress AND disorder)) AND ('parent'/exp OR parent* OR father OR patern*)
Embase	('adjustment disorder*' OR depression OR 'depression'/exp OR anxiety OR 'anxiety'/exp OR 'anxiety disorder'/exp OR 'attention deficit disorder' OR adhd OR 'behavio*r disorder*' OR 'behavior disorder'/exp) AND ('juvenile'/exp OR child* OR teen* OR adolescen* OR youth* OR juvenile*) AND ('post traumatic stress disorder'/exp OR ((posttraumatic OR (post AND traumatic)) AND stress AND disorder)) AND ('parent'/exp OR parent* OR father OR patern*) AND ('armed forces' OR military OR 'military'/exp OR army OR navy OR 'air force' OR veteran* OR 'veteran'/exp OR soldier* OR marine* OR deploy*)
PubMed	("father*"[All Fields] OR "parent*"[All Fields] OR "Parents"[MeSH Terms] OR "patern*"[All Fields]) AND ("stress disorders, post traumatic"[MeSH Terms] OR ("stress"[All Fields] AND "disorder*"[All Fields] AND "post traumatic"[All Fields]) OR "post traumatic stress disorder*"[All Fields] OR "ptsd"[All Fields] OR ("post traumatic stress disorder*"[All Fields] OR "posttraumatic stress disorder*"[All Fields])) AND (("adjustment disorder*"[All Fields] OR ("depressed"[All Fields] OR "depression"[MeSH Terms] OR "depression"[All Fields] OR "depressions"[All Fields] OR "depression s"[All Fields] OR "depressive disorder"[MeSH Terms] OR ("depressive"[All Fields] AND "disorder"[All Fields]) OR "depressive disorder"[All Fields] OR "depressivity"[All Fields] OR "depressive"[All Fields] OR "depressively"[All Fields] OR "depressiveness"[All Fields] OR "depressives"[All Fields]) OR ("Anxiety Disorders"[MeSH Terms] OR "anxiety disorder*"[All Fields] OR ("anxiety"[MeSH Terms] OR "anxiety"[All Fields] OR "anxieties"[All Fields] OR "anxiety s"[All Fields])) OR ("attention deficit disorder with hyperactivity"[MeSH Terms] OR ("attention"[All Fields] AND "deficit"[All Fields] AND "disorder"[All Fields] AND "hyperactivity"[All Fields]) OR "attention deficit disorder with hyperactivity"[All Fields] OR "adhd"[All Fields] OR "Attention Deficit and Disruptive Behavior Disorders"[MeSH Terms] OR "attention deficit disorder*"[All Fields]) OR ("behavior disorder*"[All Fields] OR "behaviour disorder*"[All Fields] OR "disruptive, impulse control, and conduct disorders"[MeSH Terms])) AND ("child*"[All Fields] OR "teen*"[All Fields] OR "adolescen*"[All Fields] OR "Adolescent"[MeSH Terms] OR "Adolescent"[All Fields] OR "youth*"[All Fields] OR "youths"[All Fields] OR "youth s"[All Fields] OR "juvenile"[All Fields] OR "juvenile s"[All Fields] OR "juveniles"[All Fields] OR "juvenility"[All Fields] OR "Adolescent"[MeSH Terms] OR "Child"[MeSH Terms] OR "Infant"[MeSH Terms]))
PubMed	('adjustment disorder*' OR depression OR 'depression'/exp OR anxiety OR 'anxiety'/exp OR 'anxiety disorder'/exp OR 'attention deficit disorder' OR adhd OR 'behavio*r disorder*' OR 'behavior disorder'/exp) AND ('juvenile'/exp OR child* OR teen* OR adolescen* OR youth* OR juvenile*) AND ('post traumatic stress disorder'/exp OR ((posttraumatic OR (post AND traumatic)) AND stress AND disorder)) AND ('parent'/exp OR parent* OR father OR patern*) AND ('armed forces' OR military OR 'military'/exp OR army OR navy OR 'air force' OR veteran* OR 'veteran'/exp OR soldier* OR marine* OR deploy*)
Web of Scienc	((TS=((adjustment disorder*) ) OR TS=((depression*)) OR TS=((depressive*)) OR TS=((depressive disorder*)) OR TS=((anxiety*)) OR TS=((attention deficit disorder*)) OR TS=((ADHD*)) OR TS=((behavior disorder*)) OR TS=("behaviour disorder") ) AND (TS=((child*) OR (teen*) OR (adolescen*) OR (youth*) OR (juvenile*)))) AND (TS=((posttraumatic stress disorder*) OR (post traumatic stress disorder*) OR (PTSD*)) AND TS=((father* OR patern* OR parent*)))
Web of Scienc	(TS=((Armed Force*) OR (Militar*) OR (Army) OR (Navy) OR (Air Force*) OR (Veteran*) OR (Marine*) OR (Deploy*))) AND (((TS=((adjustment disorder*) ) OR TS=((depression*)) OR TS=((depressive*)) OR TS=((depressive disorder*)) OR TS=((anxiety*)) OR TS=((attention deficit disorder*)) OR TS=((ADHD*)) OR TS=((behavior disorder*)) OR TS=("behaviour disorder") ) AND (TS=((child*) OR (teen*) OR (adolescen*) OR (youth*) OR (juvenile*)))) AND (TS=((posttraumatic stress disorder*) OR (post traumatic stress disorder*) OR (PTSD*)) AND TS=((father* OR patern* OR parent*))))
PILOTS	(TIABSU("child*" OR "teen*" OR "adolescen*" OR "youth*" OR "juvenile*") OR MAINSUBJECT.EXACT.EXPLODE("children") OR MAINSUBJECT.EXACT.EXPLODE("adolescents")) AND ((MAINSUBJECT.EXACT.EXPLODE("Adjustment Disorder") OR TIABSU("adjustment disorder*")) OR (MAINSUBJECT.EXACT.EXPLODE("Depressive Disorders") OR TIABSU(depression)) OR (MAINSUBJECT.EXACT.EXPLODE("Anxiety Disorders") OR TIABSU("anxiet*") OR TIABSU("anxiety disorder*")) OR (MAINSUBJECT.EXACT.EXPLODE("ADHD") OR TIABSU(attention deficit disorder*) OR TIABSU("ADHD*")) OR (MAINSUBJECT.EXACT.EXPLODE("Disruptive Behavior Disorders") OR TIABSU(behavio*r disorder*))) AND ((TIABSU("PTSD*" OR "post-traumatic stress disorder*" OR "posttraumatic stress disorder*") OR MAINSUBJECT.EXACT.EXPLODE("PTSD")) AND (TIABSU("father*" OR "parent*" OR "patern*") OR MAINSUBJECT.EXACT.EXPLODE("parents") ))
PILOTS	(MAINSUBJECT.EXACT.EXPLODE("military personnel") OR TIABSU("militar*") OR TIABSU("Arm*") OR TIABSU("Nav*") OR TIABSU("Air Force*") OR TIABSU("Veteran*") OR TIABSU("Marine*") OR TIABSU("Deploy*") OR TIABSU("Soldier*")) AND ((TIABSU("child*" OR "teen*" OR "adolescen*" OR "youth*" OR "juvenile*") OR MAINSUBJECT.EXACT.EXPLODE("children") OR MAINSUBJECT.EXACT.EXPLODE("adolescents")) AND ((MAINSUBJECT.EXACT.EXPLODE("Adjustment Disorder") OR TIABSU("adjustment disorder*")) OR (MAINSUBJECT.EXACT.EXPLODE("Depressive Disorders") OR TIABSU(depression)) OR (MAINSUBJECT.EXACT.EXPLODE("Anxiety Disorders") OR TIABSU("anxiet*") OR TIABSU("anxiety disorder*")) OR (MAINSUBJECT.EXACT.EXPLODE("ADHD") OR TIABSU(attention deficit disorder*) OR TIABSU("ADHD*")) OR (MAINSUBJECT.EXACT.EXPLODE("Disruptive Behavior Disorders") OR TIABSU(behavio*r disorder*))) AND ((TIABSU("PTSD*" OR "post-traumatic stress disorder*" OR "posttraumatic stress disorder*") OR MAINSUBJECT.EXACT.EXPLODE("PTSD")) AND (TIABSU("father*" OR "parent*" OR "patern*") OR MAINSUBJECT.EXACT.EXPLODE("parents") )))

### Inclusion criteria

The protocol for this study included only peer-reviewed publications in English. Articles were only included if published between 2000 and 2022 and focused on the study's main aim. In order for articles to be included in the review, articles had to identify paternal diagnosis of PTSD or stress related disorder. Although PTSD was not a primary diagnosis explored in children of fathers with PTSD, there is an overlap between PTSD and child mood disorders. For example, four PTSD criteria (decreased interest in activities, sleep disturbance, restricted affect, and decreased concentration) are associated with major depressive disorder. Additionally, three symptoms of PTSD (decreased concentration, irritability, and sleep disturbance) also overlap with symptoms of generalized anxiety disorder (GAD), supporting the inclusion of mood disorders as a primary focus of child psychological outcomes. The following child diagnoses were the focus of this study: adjustment disorders, mood disorders, anxiety disorders, depressive disorders, and ADHD. These diagnoses were selected based on the most prevalent mental-health diagnoses in children identified from previous studies assessing the impact of military-parental injury on mental-health outcomes in children (Hisle-Gorman and Susi 2021). Serious mental disorders (bipolar and psychosis) were not included due to the stronger biological underpinnings and complexity of these conditions. The most diagnosed mental-health disorders in children in the United States, include ADHD (9.8%), anxiety (9.4%), behavior problems (8.9%), and depression (4.4%), as identified by the Center for Disease Control (CDC), and provides additional data in support of the selected mental-health diagnosis [54]. Additionally, the child’s age range at the time of the assessment must be from 3 to 17, or the author must identify a group mean age of less than 18. Study designs were eligible for inclusion if they were prospective, cohort longitudinal, or cross-sectional. Additional references that were considered included previous systematic reviews, meta-analysis, literature reviews, book chapters, articles that primarily focused on statistical outcomes, and descriptive statistics with no quantifiable outcome.

### Exclusion criteria

Publications were excluded if they did not meet inclusion criteria. Articles were excluded if they were not peer reviewed, including dissertations, conference abstracts, and other gray literature [30, 62, 70]. Articles were also excluded if they were meta-analysis; systematic reviews; only investigated paternal PTSD as a confounder, mediator, or moderator; did not use primary data; used only secondary data; did not use a methodology that was consistent with a high quality, rigorous research; did not use a diagnostic procedure to measure the relevant exposure and outcome; included children outside of the preidentified age range and did not either stratify analyses to focus exclusively on children ages 3–17 or report a mean age that was not inside ages 3–17.

#### Outcome

The outcomes of interest were adjustment disorders, mood disorders, anxiety disorders, depressive disorders, ADHD, and behavior disorders in children. The diagnosis of PTSD was included if identified within articles that met inclusion criteria.

#### Exposure

The exposure of interest was paternal PTSD and stress-related disorders. Paternal anxiety, mood disorders, psychosis, and substance use disorders were excluded.

##### Search strategy

An electronic literature search was conducted between December 2022 and January 2023 using key words in the following databases on 15 December 2022 and 4 January 2023: PubMed, Web of Science, Embase, PILOTS, and PsycINFO. Key word searches, based on concepts required to identify articles for study inclusion, included both military and non-military terms in the identified databases.

#### Data extraction and analysis

Search findings were saved as a file and uploaded using Covidence software. Duplicate articles were removed from Endnote reference manager and Covidence. Titles and abstracts of uploaded and non-duplicate articles were reviewed based on previously identified inclusion criteria by two independent reviewers, LE and AS. Articles that met inclusion criteria underwent multiple independent cross-checks prior to final inclusion. Conflicts for article inclusion were identified, resolved between reviewers, and a final consensus was reached.

The data extraction form was developed and piloted by LE. The final data extraction form was developed in Covidence. The final data extraction form included publication details, population description for both father and children, family demographic data, relevant measurements for both father and children, covariates, study country of origin, methods, statistical findings, and relevant findings.

The results of studies included were published in a narrative synthesis. The determination of relevant characteristics in each study was based on the identification of significant results that further expanded the field of knowledge; subdomains impacting the family system; paternal-mental-health conditions comorbid with PTSD; findings confirming or opposing previous findings; prevalent data on study focus; theoretical concepts and/or potential factors associated with risks; and differences in child outcomes associated with gender, prevention, and treatment related to paternal-PTSD and mental-health outcomes in children.

#### Overview of study quality

A risk of bias assessment was conducted for all articles selected for final inclusion (see Table 2). Reference articles were used to increase proficiency in conducting article reviews to identify essential characteristics, strengths, weakness, methodological issues, design issues, and statistical results [50, 71]. The methodological assessment of bias in the included studies was primarily based on criteria drawn from the Critical Appraisal Skills Programme (CASP) checklists, the Cochrane Handbook, and the Cochrane risk-of-bias tools for both randomized and non-randomized trials. The Cochrane risk-of-bias assessment tool evaluated several domains utilizing an algorithm based on answers to individual questions. The final evaluations for bias were determined to be either “low” or “high” risk of bias [35].

**Table 2:** Risk of bias assessment for cohort studies

Covidence #	Lead Author, year	Thestudy addresses an appropriate and clearly focused issue/question	Authors use an appropriate method to answer the question	Research participants recruited in an acceptable manner	Controls selected in an acceptable way	Authors control for all the important confounding factors	The exposure accurately measured	The follow-up of subjects is long enough	Article assessed for missing data	Article assessed for missing data. If data is missing the potential that missing data led to bias in either of the most common approaches to analysis is accounted for supporting text	Outcome measures with correctly classified and or measured without error	Results precise and reported in accordance with an available, pre-determined analysis plan	Results published in a manner that does not reflect reporting only selected results	Results of this study fit with other available evidence	Other sources of bias	Totalstar rating (Maximum = 12)
16419	Klaric', 2008	*	*	*	*	–	*	*	–	*	*	–	–	–	*	7/12
16261	Fear, 2018	*	*	*	*	*	*	*	*	*	*	*	*	*	*	12/12
16251	Parsons, 2018	*	*	*	*	–	*	*	–	*	*	*	*	*	*	10/12
5936	Herzog, 2011	*	*	–	*	*	*	*	*	*	*	*	*	*	*	11/12
5130	Al-Turkait, 2008	*	*	–	*	*	*	*	–	–	*	–	*	*	*	9/12
3991	Selimbasic, 2017	*	*	–	*	–	*	*	*	–	*	*	–	*	*	9/12

*Criteria for study quality met

–Criteria for study quality not met

## Results

### Study characteristics

Six studies met inclusion criteria in this review, collectively providing data on 1,448 fathers and 1,653 children and adolescents (see Table 3). Most of the studies included used a cross-sectional design, with one longitudinal study and one cohort study. Articles primarily assessed the mental health and well-being of the entire family system, including father, mother, and children. Paternal diagnosis of PTSD or stress-related disorder was a required inclusion criterion. Articles selected for inclusion used reliable and validated scales and interviews to measure and identify characteristics of psychopathology in children, as well as individual and family functioning. Scales that originated in the Western world or primarily English-speaking countries were examined to determine if responses on translated questionnaires would yield comparable results. Researchers verified original constructs of questionnaires to ensure assessment instruments were sufficiently replicated after translation for use by foreign countries. Paternal subjects were primarily military veterans and military service members. Research articles assessed paternal PTSD. However, children were assessed for depression, anxiety, and behavioral problems, including both physical and verbal aggression. Children were also assessed for prosocial and planful behavior.

**Table 3:** Characteristics and findings of included studies

Title	Lead author, year of publication and location of study	Journal	Article published as full text or abstract	Target population/population description Fathers	Target population/pop ulation description Children	Father sample size, race, age (range and or mean and SD)	Father's diagnosis identified and or problems	Children and adolescent Sample size, race, gender and age (range and or mean and SD)	Children's diagnosis and or problems identified	Average number of children in family
Psychological problems in children of war veterans with posttraumatic stress disorder in Bosnia and Herzegovina: Cross-sectional study	Klaric, M. 2008, Bosnia and Herzegovina	Croatian Medical Journal	Full text	Veterans who were married or lived with a partner and had children	Children of war veterans	Study group n = 154, Mean age 49.3 (SD 9.1) Control group n = 77 Mean age 47.4 (SD 11.2) No distinction of gender in either group however titles within the journal indicate the population was all fathers	PTSD	Not specified. The study only included the number of children per veteran family	Developmental problems (significant within the study group) Behavioral problems Emotional problems. Measured: Structured questionnaire assessing neurotic and emotional manifestations, and behavior dysfuntion	Study grou*p* = 2 (49.4%) Control grou*p* = 2 (44.2%)
Impact of paternal deployment to the conflicts in Iraq and Afghanistan and paternal post-traumatic stress disorder on the children of military fathers	Fear, N., 2018, United Kingdom	The British Journal of Psychiatry	Full Text	UK Armed Forces fathers deployed to 2003 Iraq conflict and non-deployed comparison group, second phase service members deployed to Afghanistan with at least 1 child. Participants Served in the army (68.3%) and were non- commissioned officers (67.2%). 621 fathers have 1044 children, 87.9% have one or two children with 2.5% reporting four or more	Children between 3 and 16 with fathers who scored >= 40 on PTSD checklist	Study population n = 1,030, completed survey n = 621	PTSD	n = 1044, Males n = 524, Females n = 520, < 11 years n = 607, >/=11 n = 437	Screening for emotional and behavioral difficulties	87.9% = 1 or 2 children, 2.5% = 4 or more children
Associations of Army fathers‚ symptoms and child functioning: Within and between‚ family effects	Parsons, A., 2018, United States	Family Process	Full Text	Married couples (civilian wife, active duty husband with children	Children of fathers in active duty	n = 419 fathers, 71% non-Hispanic White, 12.9% Hispanic, 9.7% African American, Asian American 1.0%, Hawaiian or Pacific Islander 0.7%, Native American or Alaska Native 0.5%, and Multiracial 3.7% The average age of service members was 29.9 (SD = 6.02)	PTSD	Households included between 1 and 6 children, with the mean being 2.1 and the average age being 5.6 years (SD = 4.0)	Externalizing and internalizing behavior problems	Mean = 2.1, SD = 1.0
Do secondary trauma symptoms in spouses of combat-exposed National Guard soldiers mediate impacts of soldiers trauma exposure on their children?	Herzog, J. 2011, United States	Child & Adolescent Social Work	Full Text	Soldiers assigned to Midwestern Army National Guard who are fathers	Children of male Soldiers assigned to the national guard who are residing in the Soldier's/father' s home	n = 54, Black = 0, Hispanic, = 2, Caucasian = 51, Other = 1; Age range 28–53, Mean age 37.8 SD = 5.9	PTSD	Number of children not reported. Age range 2–17; oldest evaluated age 6–17, Mean age of oldest child 6, SD = 4.54; Gender of oldest female = 31, Male = 23; Race: Black = 0, Hispanic = 2, Caucasian = 52, Other = 1	Child Stress and Child behavioral problems	Not identified
Psychopatholo gical status, behavior problems, and family adjustment of Kuwaiti children whose fathers were involved in the first Gulf War	Al-Turkait, F., 2008, Kuwait	Children and Adolescent Psychiatry and Mental Health	Full Text	Fathers who completed service in the Kuwaiti Army (retired, active in Army, In battle, POWs), 187 were married and 166 wives had children	Children of father's who were Kuwait Army veterans. Defined a child as one who was still living at home	Total Assessed n = 200, married n = 187 , Mean age 37.87, SD 8.8; age range 24–71	PTSD Diagnosis Co-morbid anxiety/depress ion, internal locus of control, self-esteem, and family functioning	Total n = 489, Male Mean age = 13.6, Female Mean age 13.5; SD 5.4; Age range 6–33years	Children psychopatholo gy, behavior and family adjustment	Mothers had an average of 4.6 children as defined (SD 2.2)
Behavioral problems and emotional difficulties at children and early adolescents of the Veterans of War with post-traumatic stress disorder	Selimbasic, Z., 2016, Bosnia and Herzegovina	Medical Archives	Full Text	Fathers/Veteran of war with PTSD	Children of fathers/veteran s with PTSD	N/A	PTSD	120 school-aged children, Mean age = 12.69 (SD = 1.46), Total M/F = 50.8% vs 49.2%	Behavioral and emotional problems, levels of depression and neuroticism	2 children

Title	Lead author, year of publication and location of study	Average size of family in home with identified	Relevant paternal exposure(s), measurement instrument(s) used	Relevant child adolescent outcome(s), measurement instrument(s) used	Covariates considered	Country in which the study conducted	Aim of study	Study design	Method of recruitment of participants	Statistical analysis	Relevant findings
Psychological problems in children of war veterans with posttraumatic stress disorder in Bosnia and Herzegovina: Cross-sectional study	Klaric, M. 2008, Bosnia and Herzegovina	All participants were married with the average household having two children. The average family size of 5 was (49.4%)	CAGE Questionnaire for alcohol use PTSD-Harvard Trauma Questionnaire‚ Bosnia and Herzegovina version (HTQ-BH)	16 question survey specific to this study evaluating developmental factors, behavioral dysfunctions and emotional issues. (Assessment only conduct by fathers)	Father PTSD and Father diagnosis of alcohol use disorder father education level, employment status, economic status, father chronic health.	Other: Bosnia and Herzegovina	Assess veteran fathers with war related PTSD perceptions of psychological problems in their children.	Cross sectional study	Veterans recruited as clinic patients. Researchers obtained participants using the snowball sampling method through interviews and veteran associates	Veterans in the study group scored significantly higher on the total traumatic symptoms than the control group: Study group 2.40 (+/−) SD .057; Control Group 1.38 (+/−) SD .033, *p *< 0.0001. Children: Study Group Veterans reported that their children had higher rates of night fears Study Group 27 (17.5 Control Group 2 (2.6) Odds Ration (7.97 Confidence interval 1.84–34.48, *p *= 0.005); difficulties in school (no data reported due to lack of data) and depressive problems Study Group 23(14.9) Control 1(1.3) Odds Ration 13.34 (1.77–100.79) Children Developmental Problems Study Group Odds Ration 2.37 (1.51–3.73) *p *< 0.001; Behavioral Dysfunctions Odds Ratio 3.92 (1.53–10.03, *p *= 0.004); Emotional Problems Odds Ration 17.74 (2.49–131.10, *p *= 0.005)	Veterans with PTSD had higher perceptions of developmental, behavioral and emotional problems in their children. This includes the top two problems being night fears (17.5%) and depressive problems (14.9). Veterans with PTSD were more often of lower economic status, unemployed, and chronically ill.
Impact of paternal deployment to the conflicts in Iraq and Afghanistan and paternal post-traumatic stress disorder on the children of military fathers	Fear, N., 2018, United Kingdom	Not identified	Trauma exposure; -Strengths and Difficulties Questionnaire (SADQ), -Clinician Administered PTSD Scale (CAPS-IV). -Post-Traumatic Disorder Checklist (PCL) -Alcohol Use Disorders Identification Test	25-item Strengths and Difficulties Questionnaire (SDQ) (Assessment only conducted by fathers)	Mother's mental health and interaction with child outcome , father mental health, paternal deployment status, paternal PTSD status (as measured by CAPS), paternal reporting of symptoms of avoidance and numbing (as these symptoms are considered to be socially impairing and interfere with parenting) and childhood SDQ outcomes; child ages < 11 years and >= 11 years and child gender	UK	Examine the emotional and behavior well-being of children who fathers' are or were in the UK armed forces during the conflicts in Iraq and Afghanistan (between 2003 and 2009), the effect of paternal deployment to Iraq or Afghanistan on children from military families, the effect of paternal PTSD on children from military families, and, the specific impact of paternal avoidance and numbing symptoms on childhood emotional and behavior well-being.	Cohort study	Mail	Unadjusted Effect of paternal PTSD on children PTSD is associated with childhood hyperactivity (odds ratio (OR) 2.01, 95%CI 1.20‚3.36), prosocial difficulties (OR = 2.24, 95% CI 1.04‚4.81) and total difficulties (OR = 1.93, 95% CI 1.06‚3.51). Following adjustment for child and father demographics and father military factors, only the association with childhood hyperactivity remained statistically significant (OR = 1.86, 95% CI 1.06‚3.28) Paternal Avoidance/Numbing with childhood prosocial difficulties were statistically significant, *p* = 0.0377 Children Hyperactivity full case was significant at *p* = 0.03, prosocial full case significant *p* = 0.023 and total difficulties (SDQ) significant *p* = 0.017	Paternal deployment to Iraq or Afghanistan was not associated with childhood emotional and behavior difficulties. Paternal probable PTSD were associated with child hyperactivity. This finding was limited to boys and those under 11 years of age. Study showed that adverse childhood emotional and behavior well-being was not associated with paternal deployment but was associated with paternal probable PTSD.
Associations of Army fathers‚ symptoms and child functioning: Within and between‚ family effects	Parsons, A., 2018, United States	Married couples with children between 1 and 6 (Mean 2.1 children, SD = 1.0).	Post-Traumatic Disorder Checklist (PCL)	Behavior Problem Index (BPI) and Child Behavior Checklist (Assessment conducted by fathers and mothers)	Number of children in household and age of child, deployment status, PTSD symptoms	United States	Evaluate the link between parental PTSD and children‚ and child wellbeing by investigating within-family changes over time separately from overall differences between different families.	Longitudinal Study	Other: Participants for this study were a selected subsample of a larger randomized controlled trial testing the impact of a relationship education intervention delivered to couples by U.S. Army chaplains	Models assessed children‚ emotional and behavioral problems as rated by each parent separately. Model 1 used MOC-rated child behavioral problems PTSD, Model 2 used FOC-rated child behavioral problems, Model 3 used MOC-rated child emotional problems, and Model 4 used FOC-rated child emotional problem families, parent ratings of child emotional problems increased significantly over time, by both parents‚ reports (y100 in Models 2 (Coefficient -.001, SE 0.001, df = 330) and 4 (Coefficient 0.005, SE = 0.002, df = 330). Father PTSD rating significant increase over time (Œ≤=0.004, *p* = 0.002)	Changes in fathers‚Äô PTSD symptoms over time were associated with corresponding changes in both mothers‚ and fathers‚ reports of child behavioral and emotional problems. These within-family findings were independent from between-family effects, which showed that higher average PTSD symptomatology was associated with more overall behavioral and emotional problems for children.
Do secondary trauma symptoms in spouses of combat-exposed National Guard soldiers mediate impacts of soldiers trauma exposure on their children?	Herzog, J. 2011, United States	Not identified	PTSD checklist Military Version (PCL-M)	Child Behavior Checklist (CBCL) (Assessment conducted by fathers and mothers)	Mother psychopathology and behavior impact on children outcomes, child age and gender	United States	1. Assess the level of traumatic stress in soldiers deployed to combat from a Midwestern Army National Guard unit 2. Determine the correlation between soldier's traumatic stress and secondary traumatic stress experienced by their spouse and children. 3. Determine if secondary spousal stress symptoms mediate the secondary stress experienced by their children.	Cross sectional study	Mail	Soldier scores on the PCL-M ranged from 17 to 78 (M = 31.57; SD = 14.10). The mean for Soldier total CBCL scores was 20.27. No significant difference between Soldier and spouse CBCL total problems score on an independent samples t test. All mean comparisons and parameter estimates were tested at a minimum significance of *p*\.05. The soldier internalizing problems mean on the CBCL was 5.44 and the scores ranged from 0 to 38. Soldier externalizing problems mean on the CBCL was 6.38 and the scores ranged from 0 to 34. There was a significant relationship between combined Soldier-spouse perceptions of total problem scores on the CBCL and PCL scores (r = .470, N = 54, *p*\.000) as well as between combined soldier-spouse perceptions of total problem scores on the CBCL and STS scores (r = .441, N = 54, *p* = 0.001). There was a significant positive correlation between Soldier perceptions of child total problems on the CBCL and the PCL (r = .405, N = 54, *p* = 0.002). The correlation was moderate in strength. Soldier perceptions of child internalizing problems on the CBCL and the PCL (r = .355, N = 54, *p* = 0.008). The correlation was not significant for Soldier perceptions of child externalizing problems on the CBCL and the PCL (r = .231, N = 54, *p* =< 0.093). Soldier perceptions of child total problems on the CBCL was positively correlated with STS scores (r = .351, N = 54, *p* = < 0.009) A moderate significant correlation was also found for Soldier perceptions of child internalizing problems on the CBCL and the STS (r = .391, N = 54, *p* = < 0.003). The correlation was not significant for Soldier perceptions of child externalizing problems on the CBCL and the STS (r = .176, N = 54, *p* = < 0.202). Spouse Child. There was a significant moderate positive correlation between spouse perceptions of child total problems on the CBCL and the STS (r = .461, N = 54, *p *< 0.000). Spouse perceptions of child internalizing problems on the CBCL and the STS. The correlation was significant (r = .532, N = 54, *p*< .0000). The correlation was not significant for spouse perceptions of child externalizing problems on the CBCL and the STS (r = .240, N = 54, *p* =< 0.08). Children with fathers who scored in the high level range on the PCL averaged at the 76.7 percentile on total problems scale on the CBCL when compared to norms. These children averaged at the 72.8 percentile on internalizing problems scale and 69 percentile on externalizing problems scale on the CBCL when compared to norms. Soldier perceptions of child total problems scores on the CBCL, Soldier perceptions of child	Results suggest that immediate family members of combat-exposed Soldiers with high levels of post traumatic stress disorder (PTSD) are at risk for developing secondary traumatic stress. Secondary trauma symptoms in these spouses are a risk-increasing mediating variable between trauma symptoms in combat-exposed Soldiers and secondary trauma symptoms in their children.
Psychopatholo gical status, behavior problems, and family adjustment of Kuwaiti children whose fathers were involved in the first Gulf War	Al-Turkait, F., 2008, Kuwait	Not identified	Clinician Administered PTSD Scale (CAPS)	McMaster Family Assessment Device (FAD), only two children who were over 12 years of age (N = 281), Child Behavior Inventory (CBI) (children < 10 years and Rutter Scale A-2-parent's version (children 6–16). (Assessed by fathers and mothers)	Mother functioning and PTSD symptoms , age of children, family functioning, both parents with PTSD, degree of father trauma exposure, children in extended family homes (vs. nuclear family homes), father PTSD status and military status. Self-esteem and PTSD scores along with locus of control and anxiety. Parental age, child's age and child's level of education	Other: Kuwait	To compare the severity of symptoms of anxiety and depression, as well as behavior abnormalities, poor adaptation, and indices of poor family adjustment among the children of Kuwaiti military men, To assess the relationship of fathers' other characteristics (i.e., the prevalence of PTSD, co-morbid anxiety/depression, indices of family adjustment, locus of control and self-esteem), with indices of child psychopathology, behavior and family adjustment, on the other hand. To assess the relationship between the mothers' psychopathology. To examine the relationship between fathers'/mothers' PTSD and the children's psychopathology, behavior and family adjustment	Cross sectional study	Phone	In the final probable abnormal test scores and comorbidity for sub-scales of the three child outcome instruments, children with clinical anxiety also had clinical depression (X^2^ = 67.8, df = 1, *p* < 0.0001, in each case). Clinical depression was highly significantly associated with child's aggressive behavior (X^2^ =37.3, df = 1, *p* < 0.0001), deficient prosocial behavior (X^2^ = 9.4, df = 1, *p* < 0.002, and deficient planning behavior (X^2^ = 5.1, df = 1, *p* < 0.002. Clinical anxiety was significantly associated with child's aggressive behavior (X^2^ = 34.6, df = 1, *p* < 0.001) and deficient prosocial behavior (X^2^ = 6.1, df = 1, *p* < 0.01) -Father's combat Exposure child outcome scores. Children of POW veterans had higher anxiety, depression and abnormal behavior scores (CBI). (CBI adaptation). These trends reached significance for the following: (i) for depression: the POW group scored significantly higher than the retired and IB (*p* < 0.003); (ii) for Rutter Statements on behavior, the POW group scored significantly higher than the AIA (*p* < 0.03); and (iii) for prosocial behavior, the POW group had higher scores than the IB group (*p* < 0.006) -Family Adjustment scores. Children of retired veterans tended to have more positive adjustment scores. Family problem solving and communication (vs. the IB group) (*p* < 0.001), and for FAD Roles (vs. AIA) (*p *< 0.003)	Key Results. Children of POWs tended to have higher anxiety, depression, and abnormal behavior scores. Children whose fathers had PTSD had significantly higher depression scores. However, children of fathers with both PTSD and POW status did not have significantly different outcome scores than the other father PTSD/combat status groups. The mother's PTSD had a greater impact on the child outcome variables than the father's PTSD.
Behavioral problems and emotional difficulties at children and early adolescents of the Veterans of War with post-traumatic stress disorder	Selimbasic, Z., 2016, Bosnia and Herzegovina	94.2%, live in a 3–5 member family, while 64.2%, live in a family with two children	Harvard Trauma Questionnaire, version Bosnia and Herzegovina	Child Behavior Checklist for parents of children aged 6–18 (CBCL) Hamburg scale of neuroticism and extroversion for children and youth (HANES), Depression self-rating scale (DSRS) (Assessed by fathers only)	None were specified	Other: Bosnia and Herzegovina	Determine the exposure, manifestations of behavioral problems and emotional difficulties at children and early adolescents, whose fathers were the veterans of war demonstrating post-traumatic stress disorder symptoms	Cross sectional study	Clinic patients	Social and school conduct of children and early adolescents of the veterans of war treated for PTSD and children of the veterans of war who were not treated, statistically significant discrepancy (*p *< 0.001), as well as negative correlation (*p *< 0.01) of the symptoms of post-traumatic stress disorder of the veterans of war at HTQ and overall capabilities of their children. Positive correlation was determined in the patterns of behavior and emotional problems Internalization & Externalization of children of the veterans of war with the symptoms of fathers veterans of war (*p *< 0.01) Hamburg Neuroticism and Extroversion Scale (HANES):Statistically important discrepancy were found at neurotcism sub-scales (*p *< 0.001). Significant positive correlation was determined between neuroticism and severity of PTSD, functionality of PTSD, total PTSD (*p *< 0.01). Negative correlation was determined between sociability and severity of PTSD, functionality of PTSD, total PTSD (*p *< 0.01) Gender, group and depression level, no statistically significant discrepancy was determined (*p *> 0.05)	Children demonstrated significant behavior problems and emotional difficulties. Children showed high levels of neuroticism compared to children of fathers without PTSD. Correlation significance with the psychopathology of war veterans and their children.

### Paternal PTSD and child psychopathology

Six studies identified for inclusion specifically investigated the association between paternal PTSD and child psychological and behavioral health outcomes. Additionally, articles included in the systematic review focused solely on fathers exposed to military trauma, preventing the ability to determine if rates of child psychopathology differed between non-military fathers and military fathers. Studies found evidence to support the positive association between paternal PTSD and child or adolescent psychopathology, including a relative increase in anxiety, depression, and behavioral problems [1, 26, 34, 38, 52, 65].

Four of these articles were cross-sectional studies, three of which received high-quality ratings. One article was a cohort study, which received a high-quality rating. The cohort study conducted by Fear et al. [26] evaluated a cohort of fathers and children with a population exceeding 1,000 subjects. Researchers identified a relationship between paternal PTSD and hyperactivity in boys, and prosocial difficulties and emotional problems in both girls and boys. When examining the impact of secondary trauma and impact on family members, the cross-sectional study conducted by Herzog et al. [34] identified that family members of combat-exposed fathers with elevated levels of PTSD were at increased risk for developing secondary-PTSD symptoms. Utilizing the results of the Child Behavior Checklist (CBCL) completed by the child’s mother and father, results demonstrated that internalizing problems experienced by children was associated with secondary trauma from paternal PTSD. Additionally, spouses were identified as mediators between trauma symptoms experienced by fathers and secondary-trauma symptoms experienced by children [33]. Research by Selimbasic et al. [65] found that children exhibited elevated behavioral and emotional problems, along with increased levels of neuroticism and socially passive behavior. These findings were based on assessment tools completed by the child’s father, utilizing the Child Behavior Checklist (CBCL) to evaluate behavioral and emotional difficulties, and the Hamburg Neuroticism and Extraversion Scale (HANES) to measure personality traits.

Consistent with the relationship between paternal PTSD-symptom severity and increase rates of offspring–child psychopathology, the longitudinal study completed by Al-Turkait and Ohaeri [1], found increased rates of depression, anxiety, and increased antisocial behavior in children of former prisoners of war (POWs). This study relied on the Family Adjustment Device, Rutter A-2 Scale-Parents’ Version, and Child Behavior Inventory (CBI), which were completed by the mother and father. Additionally, children of former POWs scored significantly higher for behavioral problems compared to active service members without combat exposure and service members who experienced combat exposure but were not POWs. When exploring internalized mood problems experienced by children of father’s diagnosed with PTSD, utilizing CBI, depression was significantly associated with aggressive behavior and impulsivity of children. Anxiety was also associated with increased aggressive behavior and deficient prosocial behavior [1].

To assess emotional and behavioral difficulties in children of fathers diagnosed with PTSD, Fear et al. [26] administered the Strengths and Difficulties Questionnaire (SDQ) to the fathers. The SDQ evaluates five subscales: emotional symptoms, conduct problems, hyperactivity, peer relationship problems, and prosocial behavior. Each subscale is scored and classified as normal, borderline, or abnormal based on established, validated cut-off scores. Findings demonstrated that children of fathers diagnosed with PTSD had a higher prevalence of prosocial difficulties, conduct problems, and hyperactivity. Additionally, researchers identified higher rates of peer problems among children within the paternal-PTSD group. For girls and children older than age 11, no associations were identified between childhood difficulties and paternal PTSD. An additional finding associated with gender identified differences between boys and girls older than age 11; boys reported higher rates of hyperactivity and girls older than age 11 reported a higher prevalence of symptoms consistent with emotional problems [26].

Researchers identified a moderate correlation between paternal and maternal perceptions of the internalization of problems by children and PTSD severity as measured by the PCL and stress-trauma scores. These findings were consistent with previous research supporting the association between paternal anxiety and an increase in internalizing and externalizing behaviors in children [9, 16]. No correlation was identified between paternal and maternal reporting of externalization of problems by children as measured by the CBCL (Child Behavior Checklist) and the Stress and Trauma Score (STS) [33].

Veterans with PTSD reported high rates of traumatic events, more severe PTSD symptoms, personal dysfunction, and total traumatic symptoms. Additionally, veteran fathers diagnosed with PTSD reported higher rates of psychosocial dysfunction in their children. The structured questionnaire utilized to assess children symptoms consisted of three categories of problems: significant developmental problems, emotional problems, and behavioral dysfunction, when compared to children of veterans without PTSD [38]. Veterans reported their children experienced night fears; school difficulties, including truancy; and displayed depressive symptoms. Children of Vietnam veterans diagnosed with PTSD reported elevated levels of conflict in their families.

When exploring the relationship between paternal PTSD scores and the effects on victim’s families, both mothers and fathers reported higher rates of behavioral problems in children. Fathers with higher-rated scores rated the severity of their children’s behavior and emotional problems higher, but the significant association between father’s scores and negative child’s outcomes was only based on the mother’s rating. However, the effect of paternal PTSD on the family was significant for both fathers’ and mothers’ ratings of behavioral and emotional problems in children as measured by internalizing and externalizing scales of the Behavior Problem Index (BPI). The study demonstrated that not only do children of fathers with higher levels of PTSD symptomatology perceive their children has having more difficulties, but changes in PTSD symptoms over time are associated with changes in both mothers’ and fathers’ perceptions of their children’s functioning [52].

### Gender

No significant differences were identified when assessing levels of depression between male and females. However, one study identified that of the 21% of children who endorsed depressive symptoms, 14% were girls compared to only 7.5% of boys [65]. Findings showed that boys and girls from ages 13–15 demonstrated depressive symptoms. However, boys aged 10–12 and females aged 13–15 were identified as having higher rates of endorsing high-risk behaviors. The pattern was different for girls, for whom both internalizing and externalizing problems were associated with the degree of demoralization of the veteran’s partner. An examination of the paths of paternal PTSD to internal and externalized psychopathology by way of demoralization identified an indirect relationship between both internalization and externalization behavior, supporting the identification of an indirect impact of secondary trauma [76]. Additionally, male children and early adolescents showed more severe attention and concentration problems compared to female children and early adolescents [65]. Findings from studies measuring CBCL internalizing and externalizing scores between girls and boys found that girls had higher clinical-range scores in internalizing compared to externalizing behaviors, while boys demonstrated higher clinical scores associated with externalizing behavior [76].

### Specific aspects of PTSD diagnosis

Researchers assessed the impact of PTSD symptoms based on responses to PTSD assessment scales including symptoms identified from DSM-V criterion B to criterion E. Studies identified a statistically significant relationship between the paternal-PTSD symptom of avoidance and childhood prosocial-behavior difficulties. Additional associations were identified with peer problems for boys older than age 11 and conduct problems among boys of all ages [26]. The research literature supports the hypothesis that the numbing and avoidance symptoms of PTSD are primarily linked to the components of PTSD that result in family dysfunction; these symptoms are associated with avoiding people, conversations, and activities, as well as a loss of interest, detachment from others, and a lack of emotional response, adversely impacting children and family members [37, 43, 44]. Findings were consistent with girls, demonstrating the relationship between paternal avoidance symptoms, numbing, and prosocial difficulties.

### Severity of combat exposure

Children of POW veterans were consistently identified as having higher rates of anxiety, depression, and behavior dysfunction, as indicated by reporting higher scores on the Child Behavior Inventory (CBI) for adaptation [1]. In the case of family adjustment, children of retired veterans reported more positive adjustment scores for family problem solving and communication compared to children of fathers exposed to combat and fathers who were serving on active duty [1]. Children with fathers who were POWs but not diagnosed with PTSD had higher scores in depression, anxiety, and behavioral problems, as well as lower engagement in prosocial behavior [1].

When assessing articles that explored the relationship between the severity of paternal-PTSD scores and impact of child psychopathology, previous studies established cut-off scores for the original PTSD-Checklist (PCL) at 50 [73]. However, a study conducted by Bliese et al. [6] concluded that PCL-Military (PCL-M) cut off scores between 30 and 34 could be used to screen for PTSD among military personnel who deployed in support of operations in Iraq and Afghanistan. When analyzing the paternal association between PCL scores greater than 48 and impact on offspring, children of fathers with higher-range PCL scores reported higher scores on the CBCL to include both internalizing and externalizing problems, compared to children of fathers without positive PCL scores [33].

### Paternal level of combat exposure and military status

The literature focused on the impact of veteran PTSD and the level of veteran combat exposure on their child’s psychological health offers varied results. Some studies reported that veteran PTSD combined with an elevated level of combat exposure impacted perceived child behavior and or psychopathology, family functioning, and resulted in significantly higher levels of children reporting family conflict [13]. However, behavioral problems in children and marital-adjustment issues were primarily correlated with PTSD, rather than combat level [12, 75, 96]. Other researchers found that a veteran’s combat exposure was positively correlated with hostility and violent behavior among their children. In addition to research assessing the impact of the severity of combat exposure on child and family functioning, further evaluations focused on differences in father’s military status. Children of fathers who were retired and did not meet diagnostic criteria for PTSD reported lower symptom scores on psychopathology and abnormal behavior indices and higher levels of family adjustment. Covariates impacting child psychopathology, abnormal behavior, and family adjustment with the retired fathers without PTSD were the father’s age, and the child’s age and education. Depression and anxiety scores were also no longer significant [1].

### Relationship of paternal PTSD with partner’s mental health

Research exploring the impact of veteran-paternal PTSD and post-combat experiences on their partners identified that partners reported increased feelings of anxiety, loneliness, hostility, significant psychological distress, and somatization, as well as decreased optimism. These findings suggested that the experience of the secondary effects of paternal PTSD occurred immediately upon being exposed to their partner’s trauma symptoms [72], and that a partner’s mental health status can influence psychological outcomes in children.

Maternal trauma scores were significantly correlated with experiencing verbal abuse. Secondary trauma stress in spouses was negatively correlated with education and Soldier rank. Secondary trauma scores in spouses were positively correlated with paternal PTSD scores. Additionally, higher paternal stress and trauma symptoms resulted in increased maternal emotional distress and depressive symptoms, which is consistent with previous literature [3, 23, 24].

The mother’s PTSD status was significantly associated with child psychopathology outcomes. Children of mothers with PTSD had significantly higher scores for anxiety, depression, aggression, lower planning-behavior scores, higher scores for maladaptive behaviors, and lower family adjustment. The relationship between child and parental variables that reached a significant level were primarily associated with the mother. Child psychopathology, behavioral, and family adjustment scores were more highly correlated with the mother’s PTSD, anxiety, and depression scores compared with the father’s scores [1]. Groups of children with fathers diagnosed with PTSD and mothers without a PTSD diagnosis reported higher levels of planning behavior than children with fathers with no PTSD and mothers with PTSD [1].

Furthermore, additional literature highlights the impact that mothers diagnosed with anxiety have on child biopsychosocial outcomes. Having a mother who was the victim of domestic violence increased the probability that the child witnessed the violence, trauma, and mother’s trauma response, subsequently increasing the rate of the child developing PTSD [67].

Children exposed to domestic violence significantly increased rates of child psychological and social distress, along with conduct problems within school [64]. Animal studies support this phenomenon as demonstrated by a study of immature monkeys who observed their mother’s anxious behavior response to a threat and also adopted a fear response [48]. These findings demonstrate the relative protective impact that a mother’s mental state has on child psychological health outcomes and family functioning.

Studies exploring the potentially moderating impact of maternal trauma symptoms between paternal-PTSD symptoms in combat veterans and emotional and behavioral problems in children identified a significant reduction in emotional and behavioral symptoms in children whose mothers were not suffering from secondary trauma. These findings were based on the secondary trauma symptoms of children, self-reported by spouses, and confirmed when analyzing the mediating effect between paternal-PTSD symptoms in Soldiers and spouse perception of child internalizing problem scores on the CBCL [5].

### Deployment status

When assessing the impact of a father’s deployed status on the effects of paternal-PTSD on the family, there was no significant relationship between the father’s deployed status and the effect of his PTSD on the rating for child psychopathology. Although no significant findings were identified for the mother’s rating on child outcomes based on the father’s deployment, mothers reported higher rates of child emotional and behavioral problems [52].

### Self-report findings

All articles that met criteria for inclusion used self-reports were either completed by the child’s father or mother. As a result, this author was unable to determine if findings based on observational studies or assessments conducted by non-parents yielded different results.

### Conflicting findings

Although there is some research evidence that supports the relationship between fathers with PTSD and increased engagement in domestic violence, a study exploring the levels of domestic violence of spouses with soldiers diagnosed with PTSD found that only 3% of the 54 spouses reported scores on the victim range of the domestic violence screening tool, Hurt, Insulted, Threatened and Screaming [19]. Additional findings inconsistent with the body of literature associated with the impact of paternal-PTSD severity, identified that when controlling for combat exposure, prewar vulnerability, and harm to civilians or prisoners, there was no statistically significant impact on child internalizing and externalizing behavior [76].

Findings associated with the severity of paternal trauma exposure and impact of paternal PTSD on children are also conflicting. Some results indicated that the predictability of PTSD and the level of combat exposure account for variance in perceived child behavior problems, including increase in aggressive behavior and variance in marital adjustment [12, 29]. Additionally, partners of Vietnam veterans demonstrated significantly higher levels of somatic symptoms, anxiety, insomnia, social dysfunction, and depression. However, children of Vietnam veterans within this study showed no significant differences on measures of psychological distress compared to children of non-Vietnam veterans [74].

## Discussion

Veterans are at increased risk for a range of negative psychosocial outcomes that affect their own health and their families' stability [83, 90, 92, 95]. Compared to the general population, veterans are more likely to experience clinically significant levels of PTSD, depression, anxiety, anger, and aggression [55, 83]. They also face higher rates of substance abuse and unstable employment [90, 95], both of which can further destabilize family systems [92]. Chronic stress within these systems is linked to elevated risks for depression, developmental challenges, physical illness, and academic difficulties among children. This systematic review examined the relationship between paternal PTSD in veterans and its impact on their child and adolescent offspring.

The principal finding of this systematic review is that the severity of PTSD symptoms in fathers exposed to military trauma can be debilitating, often disrupting family functioning and placing significant strain on spouses and children [52]. Spouses of military fathers diagnosed with PTSD reported higher rates of PTSD symptoms themselves, along with higher levels of depression and anxiety symptoms [3, 23, 24]. The severity of the veteran’s symptoms has also been positively correlated with the intensity of trauma symptoms reported by spouses, highlighting the bidirectional emotional toll within the couple dynamic [34]. This ripple effect extends to children; offspring of fathers with PTSD have higher rates of depression and anxiety, behavior problems, lower engagement in prosocial behavior, and higher levels of sleep difficulty [63].

Findings within the literature identify secondary traumatization as the primary mechanism to explain the relationship between paternal PTSD and increased rates of family dysfunction and psychopathology among children and spouses [46]. Secondary traumatization is the term used to describe the experience of individuals who develop trauma-like symptoms in response to living in close proximity to trauma victims [61] or “any transmission of distress from someone who experienced a trauma to those around the traumatized individual” [28, p. 478]. Children of fathers with PTSD may be regularly exposed to symptoms such as withdrawal, irritability, anger, aggression, avoidance, and other forms of maladaptive behavior, which create a chronically stressful environment. Prolonged exposure to such stress during sensitive developmental periods can significantly increase children’s risk of developing emotional, behavioral, and psychological concerns (Agorastos et al. 2019). These findings support the assertion that trauma can be transmitted between family members through secondary exposure, and that paternal PTSD contributes to increased risk of psychopathology in children and other family members. Further research is needed to better understand the pathways of trauma transmission and to identify effective prevention and treatment strategies for affected families.

### Future research implications

The relationship between paternal PTSD and the impact on child offspring requires further exploration of the mechanism of transfer of trauma and subsequent child psychopathology. The two major themes that emerge from the impact of paternal PTSD on veterans’ families include emotional numbing and avoidance [26]. Additionally, PTSD symptoms associated with anger, combined with emotional numbing and withdrawal negatively impacts family members, including the spouse and children [57].

Although secondary traumatization has frequently been used to explain how trauma-related distress spreads from a father to children and a spouse [33, 88], evidence also supports a parenting-mediated pathway linking paternal PTSD symptoms to adverse child outcomes. PTSD related symptoms particularly irritability, anger, hyperarousal, and emotional dysregulation may manifest in the family context as hostile, coercive, or harsh parenting behaviors, which are consistently associated with greater child psychological distress. In Valentino’s research on children exposed to potentially traumatic events, child perceptions of hostile/coercive parenting were a robust predictor of child PTSD symptoms and internalizing difficulties**,** underscoring the clinical relevance of hostility and coercion as mechanisms through which caregiver trauma symptoms may shape child adjustment [69].

Consistent with this mechanism, broader evidence indicates that parental PTSD is associated with more negative parenting practices**,** including hostility and controlling behaviors, which may increase risk for child internalizing and externalizing problems [15]. Meta-analytic findings further demonstrate a reliable association between parents’ PTSD symptom severity and children’s psychological distress**,** supporting the overall pattern that higher parental PTSD symptoms correspond to poorer child mental health functioning [40]. In large epidemiologic work, parental PTSD has also been linked to offspring internalizing problems and parental aggression, aligning with the view that PTSD-related dysregulation can undermine parenting and contribute to child distress [41]. Taken together, these findings support the proposition that paternal PTSD may affect children not only through secondary trauma exposure but also through PTSD consistent parenting behaviors particularly hostility/coercion that heighten children’s vulnerability to psychological symptoms [75].

While postnatal paternal influences on children’s psychological and behavioral development are critical, a gene environment interaction framework further contextualizes the origins of psychopathology by emphasizing the dynamic interplay between biological vulnerability and environmental exposure. Methodological approaches such as twin and adoption studies**,** as well as research examining how environmental stressors shape developmental trajectories, provide robust evidence that genetic predispositions interact with family environments to influence psychological and behavioral outcomes [68].

Within this framework, paternal PTSD related behaviors may function as salient environmental stressors that interact with children’s inherited vulnerabilities, thereby increasing the likelihood of maladaptive adjustment and psychopathology [68]. Despite the absence or removal of the stress, the physiological effects of the stressor can persist for an immeasurable amount of time if not resolved [8]. If fathers are exposed to significant stress, anxiety, and trauma prior to conception, it can adversely affect the Micro ribonucleic acids (miRNA) identified in spermatozoa. This alteration in genetic material in spermatozoa may be transferred to offspring resulting in the intergenerational transfer of trauma [58]. This concept is also supported in animal studies that demonstrate the adverse effects of stress experienced prior to conception, adversely impacting paternal offspring [8]. This theoretical framework identifies the epigenetic processes involved in intergenerational effects of stress and trauma and the relationship between an individual’s lived experiences and the lasting impact these experiences have on biological factors that affect future generations [39, 77]. The intersection between paternal preconception exposure to stress, trauma and influence on future offspring demonstrates a critical area of research that requires further exploration.

**Fig. 1:**
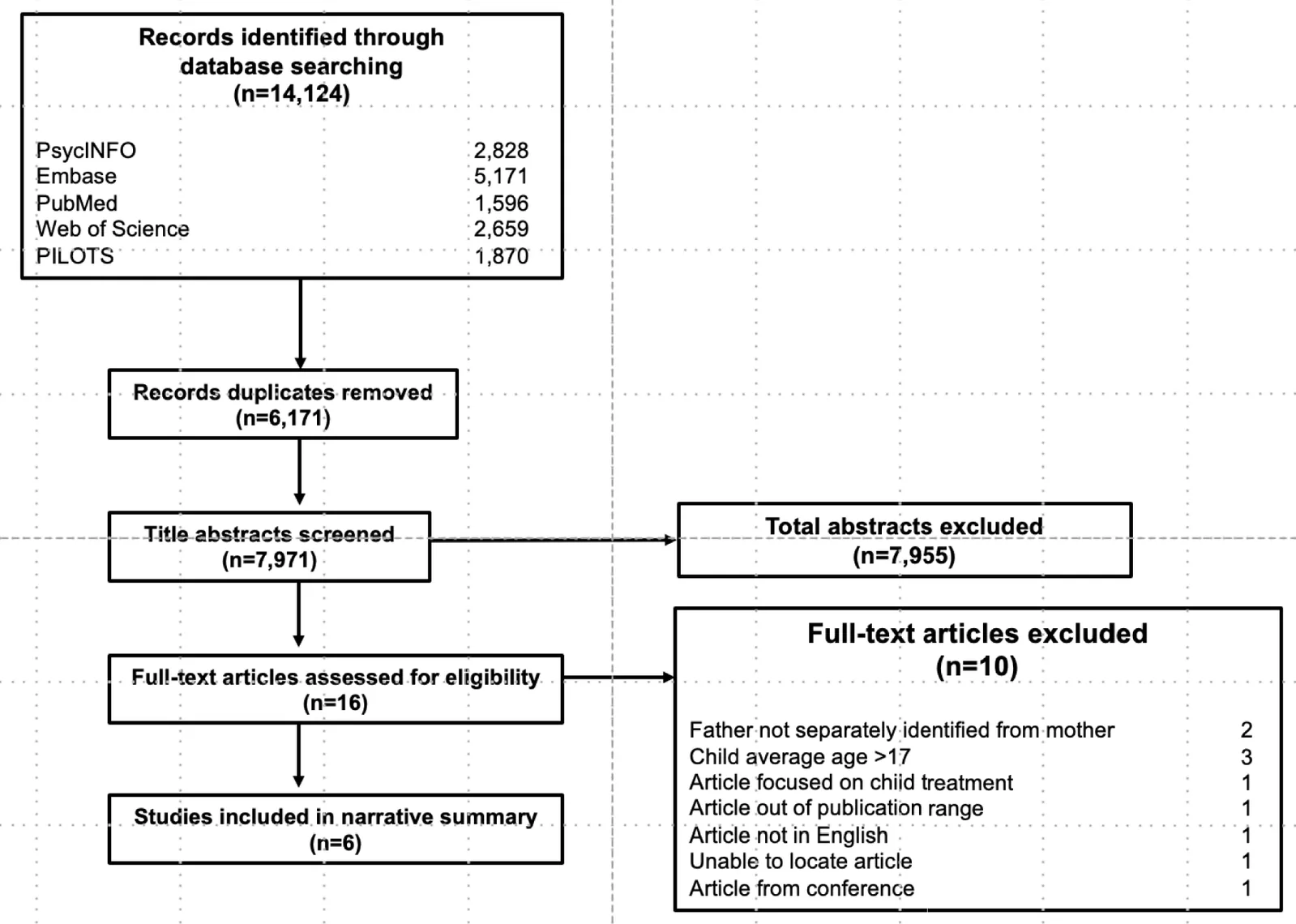
Flowchart of primary study selection

Based on the influence of paternal PTSD on the entire family, future prevention and treatment approaches must target the family system and relationships to address child and family dysfunction. The withdrawn father is often unresponsive or unavailable to attend to their child’s needs due to debilitating trauma symptoms. Children of fathers with PTSD have difficulty having their emotional and physical needs met [69]. The traumatized paternal figure will require treatment to address PTSD symptoms that influence parental behavior and interactions with family members. The evidence demonstrating adverse outcomes that impact both children and spouses in families with paternal figures diagnosed with PTSD sheds light on the need for increased exploration of prevention and treatment programs that target paternal veterans, additional caregivers, and improve the well-being of the entire family system (Fig. 1).

### Limitations

This systematic review was conducted based on PRISMA guidance reflecting the highest standard of reporting methodology to identify, select, appraise, and synthesize research [49]. In addition to using guidance from PRISMA, guidance from the Cochrane Handbook provided a descriptive and comprehensive approach to completing a systematic review [20]. Despite using identified systematic review resources and guidance, our review identified several limitations.

One of the primary limitations was the strict age criteria for children, which was limited to ages 3–17 or an average age of less than 18 of all paternal-offspring research participants. The variability of age ranges identified in studies excluded young adults, limiting the number of articles identified for assessment. Articles that met age requirements for inclusion did not universally separate findings of paternal offspring into groups of young children and adolescents. Based on evidence demonstrating psychological, neurological, and social developmental differences between young children and adolescents Bundy et al. [10], future research analysis must distinguish between age groups, including conducting statistical analysis to determine if quantifiable statistical differences exist between early-childhood and adolescent psychological and behavioral outcomes. Several of the articles exploring the relationship between children of war veterans and the war veterans, children of holocaust victims and these victims, or children of other potential traumatizing events experienced by fathers and these fathers included adult children [21, 53, 78]. These articles did not meet inclusion criteria but provided critical findings associated with the impact of paternal PTSD on child mental and behavioral health outcomes.

The second limitation of this systematic review is that articles that met inclusion criteria only explored combat-related trauma experienced by military service members and military veterans. Although military personnel are at increased risk to experience trauma associated with combat exposure, military combat is only one subcategory of trauma out of many different mechanisms of trauma exposure. No identified studies compared paternal exposure to trauma outside of military combat exposure. Different forms of trauma exposure—for example, trauma associated with sexual assault, emotional betrayal, physical abuse, natural disasters—may have a different impact on child offspring and family members [66]. Further studies are needed to explore the impact of paternal non-combat related trauma and the impact on child offspring and family.

An important limitation of this systematic review is the limited examination of paternal PSTSD as a mediating or moderating variable in the association between paternal military exposure and child mental health outcomes. Although prior research has demonstrated significant associations between parental trauma exposure and adverse psychological and behavioral outcomes in children [27, 40], relatively few studies have empirically tested whether these effects are transmitted indirectly through paternal PTSD symptomatology or vary as a function of symptom severity, chronicity, or specific symptom clusters. Evidence suggests that parental PTSD may influence child adjustment through disrupted parenting practices, emotional availability, and broader family functioning [17, 22]; however, these pathways were rarely modeled explicitly in the studies included in this review. Consequently, the absence of mediation and moderation analyses constrains the review’s ability to clarify mechanisms of intergenerational risk transmission, distinguish direct exposure effects from symptom-driven processes, and account for heterogeneity in child outcomes. As a result, findings should be interpreted as primarily associational rather than explanatory, underscoring the need for future longitudinal and theory-driven research that explicitly incorporates paternal PTSD as a mechanistic variable in models of child mental health outcomes.

An additional limitation was associated with the small number of studies identified and limited reporting of the number of children participating in these studies. Only three of the six studies that met inclusion criteria identified the exact number of children included in the study. Failure to identify the number of children may impact the researcher’s ability to detect differences between groups of children with fathers with PTSD and children with fathers without PTSD.

The final limitation was associated with the identification of only one article that examined the mediating impact of maternal PTSD and other forms of psychopathology on child psychological and behavioral health outcomes. The lack of research literature demonstrating the moderating impact of the mother’s mental health when analyzing the impact of the father’s PTSD results in difficulty in distinguishing whether the relationship between the father’s PTSD and child psychopathology was influenced by the mothers’ mental health, as supported by findings published by Herzog et al. [33].

### Conclusion

The findings of this systematic review demonstrate the relevancy of paternal PTSD and the impact on child and adolescent mental and behavioral health outcomes. Additionally, these findings show the impact of paternal PTSD not only on children but on the entire family system, including spouses of fathers with PTSD. These findings highlight the need to conduct further research to distinguish between the impact of paternal PTSD on young children and adolescents; further identify the mechanism of influence associated with paternal PTSD, child offspring and family system; further explore the maternal moderating impact of maternal psychological health of children; and identify prevention and treatment modalities to address the adverse outcomes linked to paternal PTSD and child psychopathology.

## Data Availability

No datasets were generated or analysed during the current study.
